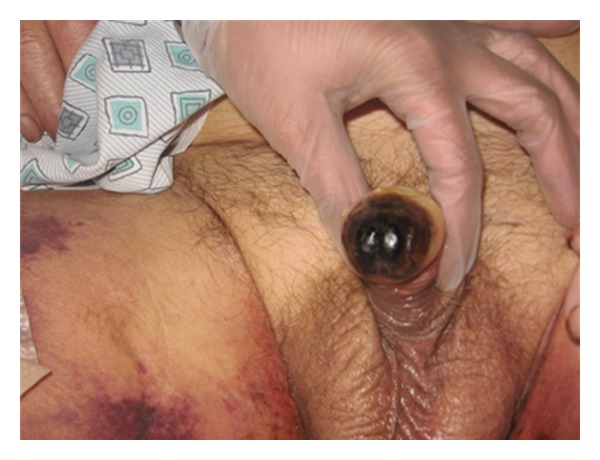# Calciphylaxis: Temporal Artery Calcification Preceding Widespread Skin Lesions and Penile Necrosis

**DOI:** 10.1155/2012/309727

**Published:** 2012-05-30

**Authors:** Manzoor A. Shah, Michael W. Roppolo

**Affiliations:** Division of Internal Medicine, Baton Rouge General Hospital, 3600 Florida Boulevard, Baton Rouge, LA 70806, USA

## Abstract

Temporal artery calciphylaxis has rarely been described in chronic kidney disease patients on dialysis. We report a case of 72-year-old Caucasian man with multiple comorbidities and end-stage renal disease on dialysis who presented with temporal artery calcification leading to bilateral loss of vision followed by extensive skin lesions including one on glans penis. While on peritoneal dialysis, he developed anterior ischemic optic neuropathy, had no improvement on high dose steroids, and temporal artery biopsy showed marked calcification without any evidence of vasculitis. Few weeks later on hemodialysis, he developed widespread cutaneous lesions on extremities and penile necrosis with skin biopsy revealing calciphylaxis. On literature review of calciphylaxis in chronic kidney disease, we found only four cases of temporal artery calciphylaxis leading to anterior ischemic optic neuropathy and blindness. We believe this is the first case in which the rare temporal artery calciphylaxis and the uncommon penile necrosis are being described together. The objective is to emphasize the need to recognize this condition early in the CKD patients on dialysis presenting with visual symptoms as the different treatment strategies may help prevent complete loss of vision and also modify or prevent a full blown calciphylaxis.

## 1. Introduction

Calciphylaxis or calcific uremic arteriolopathy (CUA) is a rare condition that occurs in chronic kidney disease (CKD) stage 4 or 5 with secondary hyperparathyroidism though it has been described in few nonrenal conditions as well. With pathogenesis poorly understood it has high morbidity and a 6-month mortality rate of about 80% [1, 2]. It occurs in 1–4% of dialysis patients usually involving areas of lower extremities with high adipose tissue like thighs and buttocks [3]. Lesions appear as violaceous painful indurated nodules, plaques, necrotic eschars, ulcerations, and dry gangrene. Diagnosis is based on clinical signs and symptoms and biopsy of the lesion by demonstrating calcification in the vascular media, intimal hyperplasia, inflammation, obliterative endovascular fibrosis and microthrombi in small- and medium-sized vessels of skin, and subcutaneous tissue leading to necrosis of dermal, subdermal, and adipose tissue [4].

## 2. Case Report

A 72-year-Caucasian man was referred to our Long Term Acute Care (LTAC) Hospital for continuation of hemodialysis and aggressive wound care. He had a PMH of hypertension and diabetes mellitus diagnosed at the age of 59 when he had aortic valve replacement (AVR) with a St Jude's valve for aortic stenosis and had been on coumadin since then. He also had atrial fibrillation (AF) since 2005 followed few years later by congestive heart failure (CHF) with right-sided heart failure being more pronounced than the left, severe tricuspid regurgitation, pulmonary artery pressure of 68 mm Hg, concentric left ventricular hypertrophy, and a LVEF of 53%. Also had diabetic complications of background retinopathy and nephropathy with stable Creatinine (Cr ~1.7 mg/dL) for several years. In July 2011, the patient started having problems with volume overload with swelling of lower extremities and ascites. His cardiologist tried outpatient diuresis with metolazone and bumetanide while balancing it with his degree of CKD but not with much success. Two months later, he needed to be admitted to the hospital with worsening volume overload, weeping leg edema, massive ascites, and declining renal functions with a BUN/Creatinine of 97/2.0 mg/dL. Diuretics were used intravenously without much effect again. He was planned for continuous ambulatory peritoneal dialysis (CAPD) and had a Tenkchoff PD Catheter put in laproscopically. In the meantime, hemodialysis was given for three weeks before starting CAPD at low volume exchanges and 5 times per day at his home. His medications included bumetanide, cholecalciferol, calcium acetate, coumadin, iron, and lantus. In December 2011, he developed sudden loss of vision in his left eye. His ophthalmologist found a left optic disc edema, venous dilatation, choroidal hypoperfusion, and a decreased visual acuity (VA) of 20/200 in his left eye and 20/40 in his right eye. He was diagnosed to have anterior ischemic optic neuropathy (AION). Three days later, he developed decreased vision in the right eye with VA of 20/80. Both optic discs were edematous with left being more than the right. The patient was started on high dose steroids and admitted to the hospital for temporal artery biopsy which revealed large coarse marked calcifications in the muscular artery with no evidence of vasculitis. Steroids did not make any improvement and were subsequently stopped. Next eye exam a week later showed a VA of 20/200 in both eyes due to AION. During the hospital stay, his peritoneal dialysis (PD) volume exchanges were increased to 2 liters per exchange.

Four weeks later, he was again admitted to the hospital for falls, and worsening uremia. His lab work showed a BUN 69 mg/dL, creat 6.3 mg/dL, GFR 9, calcium 7.9 mg/dL, phosphorus 6.6 mg/dL, calcium phosphate product 51.48 mg/dL, parathormone (PTH) 164 pg/mL, ALP 636 IU/L, Hb 10.1 g/dl, and Hct 30.2%. He had been noncompliant with PD exchanges doing only a couple of exchanges per day. Hemodialysis (HD) was started through a left IJ Vascath, and a right brachiocephalic AV fistula was created. He developed fiercely spreading wide areas of indurated skin plaques, woody in feel and dusky in color at multiple sites on thighs, legs, arms, and glans penis with two small black eschars on thighs. After he was stabilized on HD, he was transferred to LTAC for continuation of hemodialysis and aggressive wound care. He had debridement of left thigh wound, and biopsies were taken from both thighs which revealed calciphylaxis with prominent calcification of the small vessels in the subcutaneous fat accompanied by ischemic necrosis and ulceration of the overlying epidermis.

**Figure 1:**
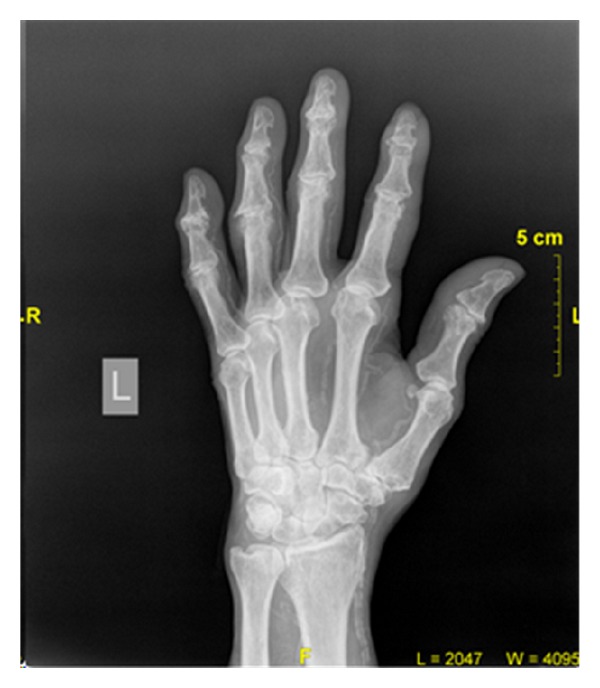


The patient developed sepsis with low-grade fever, high WBC count, neutrophilia, and altered mental status. Urinalysis was normal, blood cultures were negative but wound culture grew methicillin sensitive Staphylococcus aureus (MSSA), and antibiotics were given per sensitivity. His X-ray chest showed pleural thickening and calcification just above left hemidiaphragm and X-rays of extremities showed extensive vascular calcification (Figure 1).

**Figure 2:**
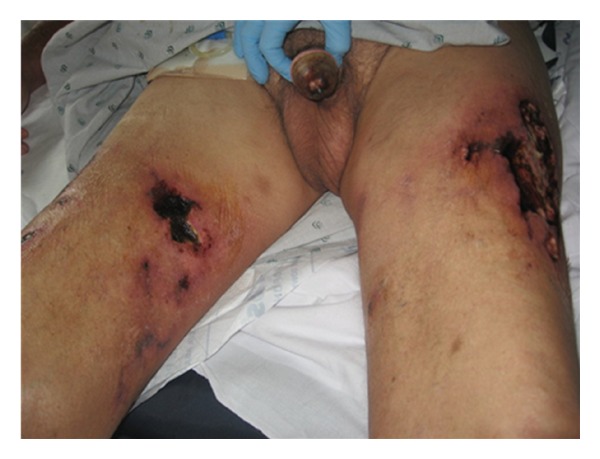


Serum PTH increased to 374 pg/mL with vitamin D <8.0 pg/mL. Hemodialysis frequency per week was increased, and the duration per session was increased to four hours. Calcium acetate was stopped and replaced by sevelamer—a noncalcium phosphate binder. He continued his other medications including Cholecalciferol and Coumadin. Wound care was carried out by a physician-led dedicated wound care team. After debridement the wound on the left thigh refused to heal with even further blackening of wound margins. Antibiotics were broadened to cover gram positive cocci, gram negative rods and anaerobes for continuing sepsis. However, his calciphylaxis worsened with black eschars developing over wide areas of indurated skin lesions and glans penis becoming gangrenous (Figures 2 and 3).

Clinically, the patient took another turn for worse when he developed coagulopathy with prolonged INR and malena.

With unabating calciphylaxis, nonhealing wounds, sepsis, coagulopathy, and gastrointestinal bleeding the patient died within ten weeks of presentation with temporal artery calciphylaxis causing blindness and six weeks of widespread skin lesions.

## 3. Discussion

Treatment of calcific uremic arteriolopathy is often unsuccessful due to pathogenesis being unclear and diagnosis frequently delayed or missed. With increasing number of surviving patients on dialysis and high prevalence of CKD, more cases of calciphylaxis are being recognized now. Risk factors include high serum phosphate level with increased calcium phosphate product, secondary hyperparathyroidism, and use of calcium containing phosphate binders, use of calcitriol, diabetes mellitus, obesity, low albumin, Coumadin therapy, female gender, and Caucasian race [2, 5].

In our case, noncompliance with peritoneal dialysis most likely led to hyperphosphatemia with a modest increase in CaP04 product and secondary hyperparathyroidism. When the patient first presented with temporal artery calcification leading to AION, the diagnosis of calciphylaxis was not considered because of this rare complication occurring in isolation without skin lesions. However, few weeks later it became apparent when the widespread skin lesions appeared like a wildfire with unrelenting course leading to cutaneous eschars and penile gangrene with skin biopsy confirming the diagnosis. Coumadin therapy most likely rendered additional risk to our patient and probably aggravated calciphylaxis. Coumadin in animals has been shown to induce vascular calcification by preventing activation of matrix gla protein (MGP) in the vessel wall. MGP is a vitamin K dependent protein, which becomes biologically active after carboxylation and prevents vascular calcification. However, the relationship of Coumadin to vascular calcification in humans is not fully known [6, 7]. Considering the benefit/risk ratio of anticoagulation in our patient with AVR, Coumadin was continued. 

Cornerstone of therapy is to keep serum phosphate <6 mg/dL and a CaPo4 product <55 mg/dL which we obtained by changing calcium-based phosphate binder to sevelamer and performing hemodialysis with a low-calcium dialysate [8]. Debridement of the wound in calciphylaxis is controversial and generally not advisable and in our case the debrided wound refused to heal. On account of his advanced debility patient was not considered for parathyroidectomy. Pharmacotherapeutic interventions like sodium thiosulfate therapy, hyperbaric oxygen therapy or cinacalcet were not used due to limited anecdotal evidence and his moribund condition [9, 10]. Sodium thiosulfate a potent antioxidant that also increases the solubility of calcium deposits, has been reported to lead to marked improvement of calciphylaxis [11]. Hyperbaric oxygen therapy has been used with some success in calciphylaxis with local tissue hypoxia in wounds. It restores tissue oxygen to normal or above normal level thereby enhancing collagen production, fibroblast proliferation, and angiogenesis [12].

Calciphylaxis of temporal artery in ESRD leading to nonarteritic AION and blindness has rarely been described and on review of literature we found only 4 cases reported [13–16]. Penile necrosis due to calciphylaxis is quite uncommon in ESRD as penis has a rich blood supply from its dorsal and deep arteries and also from urethral artery [17–19]. Its occurrence in the context of ESRD, diabetes, and dialysis carries a high mortality. We believe this is the first case of CUA in whom temporal artery calciphylaxis and penile necrosis are being reported together.

Temporal artery calciphylaxis in CKD patients on dialysis presenting with symptoms of visual loss needs to be recognized early as the different treatment strategies may help prevent complete loss of vision and also modify or prevent a full blown calciphylaxis—a condition known to have very high morbidity and mortality.

## Figures and Tables

**Figure 3:**